# Selection against spermatozoa with fragmented DNA after postovulatory mating depends on the type of damage

**DOI:** 10.1186/1477-7827-8-9

**Published:** 2010-01-31

**Authors:** Juan D Hourcade, Miriam Pérez-Crespo, Raúl Fernández-González, Belén Pintado, Alfonso Gutiérrez-Adán

**Affiliations:** 1 Dpto. de Reproducción Animal y Conservación de Recursos Zoogenéticos, INIA, Ctra de la Coruña Km 5.9, Madrid 28040, Spain; 2 Centro Nacional de Biotecnología, CSIC. C/Darwin 3 Madrid 28049, Spain

## Abstract

**Background:**

Before ovulation, sperm-oviduct interaction mechanisms may act as checkpoint for the selection of fertilizing spermatozoa in mammals. Postovulatory mating does not allow the sperm to attach to the oviduct, and spermatozoa may only undergo some selection processes during the transport through the female reproductive tract and/or during the zona pellucida (ZP) binding/penetration.

**Methods:**

We have induced DNA damage in spermatozoa by two treatments, (a) a scrotal heat treatment (42 degrees C, 30 min) and (b) irradiation with 137Cs gamma-rays (4 Gy, 1.25 Gy/min). The effects of the treatments were analyzed 21-25 days post heat stress or gamma-radiation. Postovulatory females mated either with treated or control males were sacrificed at Day 14 of pregnancy, and numbers of fetuses and resorptions were recorded.

**Results:**

Both treatments decreased significantly implantation rates however, the proportion of fetuses/resorptions was only reduced in those females mated to males exposed to radiation, indicating a selection favoring fertilization of sperm with unfragmented DNA on the heat treatment group. To determine if DNA integrity is one of the keys of spermatozoa selection after postovulatory mating, we analyzed sperm DNA fragmentation by COMET assay in: a) sperm recovered from mouse epididymides; b) sperm recovered from three different regions of female uterine horns after mating; and c) sperm attached to the ZP after in vitro fertilization (IVF). Similar results were found for control and both treatments, COMET values decreased significantly during the transit from the uterine section close to the uterotubal junction to the oviduct, and in the spermatozoa attached to ZP. However, fertilization by IVF and intracytoplasmatic sperm injection (ICSI) showed that during sperm ZP-penetration, a stringent selection against fragmented-DNA sperm is carried out when the damage was induced by heat stress, but not when DNA fragmentation was induced by radiation.

**Conclusion:**

Our results indicate that in postovulatory mating there is a preliminary general selection mechanism against spermatozoa with low motility and fragmented-DNA during the transport through the female reproductive tract and in the ZP binding, but the ability of the ZP to prevent fertilization by fragmented-DNA spermatozoa is achieved during sperm-ZP penetration, and depends on the source of damage.

## Background

Sperm DNA damage is gaining interest as a potential cause of infertility, and it may be initiated by a wide range of causes: drugs, chemotherapy, radiation therapy, cigarette smoking and environmental toxins, genital tract inflammation, testicular hyperthermia, varicoceles, hormonal factors, etc (Reviewed in [1]). The normality of sperm nuclear DNA plays a critical role in mammalian fertilization and subsequent embryonic development. It has been demonstrated that sperm cells with damaged or fragmented-DNA can fertilize oocytes *in vitro *[2]. Some authors consider that this also happens *in vivo *[3] and that highly motile mouse sperm did not differ in types and frequencies of chromosomal abnormalities from those not selected for motility [4]. However, it has been reported in humans that swim-up selection based on sperm motility excludes many sperm with ultrastructural evidence of apoptosis, as confirmed by TUNEL analysis [5]; also there is a correlation between sperm motility and sperm chromatin structure assay (SCSA) parameters [6]; and that sperm DNA fragmentation affects sperm motility and fertilization rates [7]. It has been reported *in vivo *that the likelihood of boar spermatozoa with unstable chromatin to reach and to fertilize the oocyte is very low [8]. There is evidence suggesting that the journey of the sperm cells from the site of deposition to the site of fertilization is both dynamic (by the sperm and the female tract) and highly complex [9]. Passage of sperm through the female reproductive tract is regulated to maximize the chance of fertilization and to ensure that sperm cells with normal morphology and vigorous motility will be the ones to succeed [10].

Regardless of the large number of spermatozoa in an ejaculate, only a minority are able to meet the stringent requirements needed to fertilize the oocyte. This reduction should be due to the presence of mechanisms within the female reproductive tract that act as checkpoints for the selection of fertilizing spermatozoa. The three principal mechanisms are (a) the female reproductive tract microenvironment and fluids molecules; (b) the sperm-oviduct (caudal isthmus) interaction (a pre-ovulatory mechanism); and (c) the sperm-zone pellucida interaction (a post-ovulatory mechanism) [10]. It is accepted that adhesion to the isthmus plays a key role in sperm selection and storage of sperm until ovulation. However, mating after ovulation impedes sperm attachment to the oviduct, reducing the female sperm selection mechanisms. In this case, only the sperm-zona pellucida (ZP) interaction may produce selection of the spermatozoa. In fact, it has been suggested that in humans, the binding of spermatozoa to ZP selects spermatozoa endowed with progressive motility, normal morphology and chromatin structure [11], and may also discriminate against spermatozoa with numerical chromosomal aberrations [12]. It has been reported that *in vitro*, sperm with single stranded or denatured DNA bind less or do not bind at all to the ZP [13]. In pigs, spermatozoa with stable chromatin are more likely both to bind to the oviduct and to traverse the reproductive tract *in vivo *[8], ultimately reaching the oocytes and penetrating the zona pellucida. Since the female reproductive tract does not assess the sperm DNA quality directly, the selection has to be based on sperm phenotype and function [14,15].

It is not known whether and to what extent chromatin fragmented sperm contributes to the fertilization process *in vivo*, where selective barriers of the female tract have to be overcome before the sperm meets the oocyte This is the first study to investigate in a post-ovulatory mating model, the selection mechanisms that operate in nature to find out if these mechanisms can discriminate the quality of spermatozoa DNA regardless of the origin of damage.

## Methods

### Experimental design of the in vivo experiments

All experimental procedures were approved by our Institutional Review Board according to the Guide for Care and Use of Laboratory Animals as adopted by the Society for Study of Reproduction. Mice were kept on a 14L:10D light cycle. CD1 female mice (8-10 wk old) were paired with male mice of the same strain to allow mating. Adult males CD-1 (Harlan, Oxon; UK), 12 weeks old, were used for the heat stress (n = 8) and γ-radiation (n = 9) experiments. Scrotal heat stress was realized as previously described [16]. Briefly, males were anesthetized with an intraperitoneal injection of 0.1 ml/10 g body weight of a solution containing 10 mg of ketamine and 1 mg of xylazine per ml. A cotton string was loosely applied around the scrotum to avoid retraction of the testes to an abdominal position, without constraining testicular vascularization. The males were then passed through a hole in a polystyrene "raft" up to the mid-body level, so that they floated on the water surface with their lower body and testes immersed in water at 42°C for 30 min. After returning to their cages, the animals were placed on a warm mat (25°C) to maintain their body temperature until they had fully recovered from the anesthesia. Control males (n = 5) were also anesthetized and placed in a water bath at 33°C for 30 min and recovered in the same way [16].

**Figure 1:**
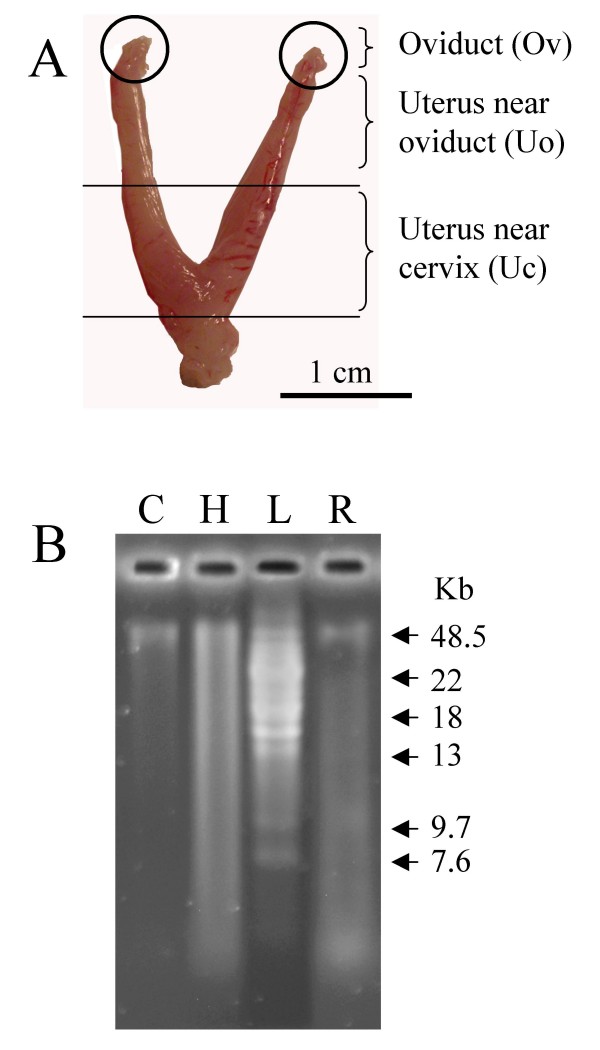
**(A) Spermatozoa analysed were recovered from three different sections as described in Material and Methods**. (B) Analysis of ssDNA breaks on DNA from control (C), heat stressed (H) and γ-radiated spermatozoa (Γ). L: ladder.

The γ-radiation was applied in mice as it has been described [17] using a ^137^Cs irradiator, adjusting dose to 4 Gy with a dose rate 1.25 Gy/min. Five control males were sham irradiated. Sperm and mating experiments were realized 21-25 days after heating and γ-radiation. For the mating experiments, each male was caged with one female (CD-1) in oestrus stage as they were chosen at evening of day before [18]. Fertilization is thought to occur *in vivo *at midnight to early morning of embryonic day (E0); this stage appears to correspond to the period 7-12 h after hCG treatment. For this reason in our post-ovulatory mating mice model, oocytes were fertilized 6-8 hours after ovulation. Two hours after caging the female with the male, females with vaginal plug were selected, and sperm were recovered form three regions of the female reproductive tract (Fig. 1A). Each uterine horn was cut at its cervical end, the uterotubal junction and a middle point obtaining 3 different regions as previously described [19]: the isolated oviduct (Ov), the proximal uterine region (Uo), close to the uterotubal junction (UTJ), and the distal uterine region (Uc), close to the cervix. Another group of females with vaginal plug was allowed to follow pregnancy until day 14, when they were slaughtered and number of embryos and resorption sites was determined.

Sperm counts from non-treated and treated males, collected from vas deferens, as well as sperm counts from different uterine sections were performed using a Bürke hemocytometer. Motility was determined by loading a sperm sample onto a pre-warmed (37°C) slide and placing it on the heated (37°C) microscope stage. Percentages of motile spermatozoa were assessed by subjective analysis of two different observers in at least 3 different fields. Since no statistical differences were found among data from the 2 control groups (sham irradiated and submitted to 33° water bath), we combined them into a single control group for further comparisons.

### Experimental design of the *in vitro *experiments

*In vitro *fertilization (IVF) was performed as described in [20]. Oocytes were collected from superovulated CD1 female mice (8-10 wk old). Superovulation was induced by intraperitoneal injections of 7.5 IU of equine chorionic gonadotropin (eCG; Intervet, Boxmeer, Holland), followed 48 h later by 5 IU of human CG (hCG; Lepori, Farma-Lepori, Barcelona, Spain) [21]. Cumulus-oocytes-complexes (COCs) were obtained from the ampulla and cumulus cells were removed by incubation in hyaluronidase in M2 medium. Groups of. 20-30 oocytes were placed in each fertilization drop of 500 μl of Human Tubal Fluid medium (HTF) supplemented with BSA and covered with mineral oil. Sperm was collected from adult CD-1 males subjected to scrotal heating and γ-radiation as described for the in vivo experiments. The males were killed by cervical dislocation 21-25 days after treatment. Spermatozoa collected from the cauda and the vas deferent were incubated in HTF drops supplemented with BSA, during 30 min at 37°C with 5% CO_2 _to achieve capacitation. After incubation, 10-15 μl of the sperm suspension were collected from the peripheral part of the capacitation drop with a pipette tip. This suspension was transferred into the fertilization drop and co-incubated with the oocytes in HTF medium (8 × 10^5 ^motile spermatozoa/ml) for 5 hours. Then, oocytes were washed three times and placed in culture drops of potassium simplex optimized medium with amino acids (KSOMaa). Cleavage rate was assessed at 24 hours post insemination and embryo culture was followed to the blastocysts stage.

Intracytoplasmatic sperm injection (ICSI) was performed as described in [22]. Briefly, metaphase II oocytes were collected from 6- to 8- week-old female mice superovulated and cumulus cells were removed with the same procedure used for IVF. Oocytes were washed and maintained in KSOMaa at 37°C in a 5% CO_2 _in air atmosphere until use. Fresh sperm samples from heat stressed, γ-radiated or control CD1 mice were used to perform ICSI. Sperm was diluted 1:5 with M2 medium containing 10% polyvinyl-pyrrolidone (PVP) to decrease stickiness (1 part sperm: 5 parts M2+PVP). ICSI was performed in M2 medium at room temperature. The ICSI dish contained a manipulation drop (M2 medium), a sperm drop (sperm solution in M2/10% PVP), and an M2/10% PVP needle cleaning drop. Injections were performed with a PMM-150 FU piezo-impact unit (PrimeTech, Tokyo, Japan) and Eppendorf micromanipulators (Hamburg, Germany) using a blunt-ended mercury-containing pipette with 6 to 7 mm of inner diameter [23]. The head of the fresh sperm cell was separated from the midpiece and tail by applying 1 or more piezoelectric pulses. Groups of 10 oocytes were injected with individual sperm heads. After 15 minutes of recovery at room temperature in M2 medium, surviving oocytes were washed 3 times in equilibrated KSOMaa and returned to mineral oil-covered KSOMaa drops and cultured at 37°C in a 5% CO_2 _air atmosphere. Cleavage was assessed at 24 hours post sperm injection and cleaved embryos were cultured *in vitro *until blastocyst.

### Collection of sperm samples from the *in vitro *fertilization experiment for DNA fragmentation analysis

Two hours after IVF all sperm bound to the surface of ZPs were removed by repeated vigorous aspiration with a narrow pipette with an inner diameter slightly smaller than the oocyte in a 35 mm-dish with 30 μl Phosphate Buffered Saline (PBS) supplemented with 0.5% Bovine Serum Albumin (BSA). Since only a small number of spermatozoa was bound to ZP in some experimental groups, 25 oocytes were used for each one. DNA damage of removed ZP-bound spermatozoa and spermatozoa retrieved from different uterine sections and males (control, heat stressed and γ-radiated) as described in *in vivo *experiment (see above) were assessed by single-cell gel electrophoresis (SCGE or Comet assay), following the protocol described in [24]. Analysis of the shape and length of "comet" tail, just like the DNA content in the tail, gives an assessment of DNA damage. Briefly, 30 μl-sperm suspension (10000 spermatozoa/μl each sample except oviduct samples in which concentration was 100 spermatozoa/μl) was diluted in low-melting-point agarose (70 μl, 1% w/v; LM-2, Hispanlab, Madrid, Spain). A 100-μl mixture of sperm-agarose was immediately pipetted onto agarose-coated slides (1% w/v normal-melting-point agarose). Samples were immersed in ice-cold lysing solution (Trevigen) supplemented with 40 mM dithiotreitol and proteinase K (200 μg/ml). Incubation was performed during 1 h at 37°C. After this step, slides were rinsed in distilled water three times (5 min each time) and incubated in electrophoresis neutral buffer (Tris-borate-EDTA, pH 8) for 20 min. Electrophoresis was then performed at 25 V and 300 mA for 7 min. Following electrophoresis, the slides were neutralized with Tris-HCl buffer (pH 7.4) for 5 min and rinsed in distilled water. Samples were stained with SYBR Green (Trevigen) and analyzed under an epifluorescence microscope (Optiphot-2; Nikon). Comets were analyzed using specialized SCGE analysis software (CometScore, freeware version; TriTek Corp). Tail length (pixels, px) and tail moment (arbitrary units, A.U.) were recorded in 100 cells per sample. Both parameters allowed us to describe extension of DNA damage. Tail length indicates the distance migrated. Tail moment is the product of the tail length and the fraction of DNA in the tail, calculated by the analysis software. It was considered as an indirect measure of the size of the fragments generated. In order to evaluate DNA damage in spermatozoa in terms of single strand breaks, we used alkaline gel electrophoresis as previously described [25]. Briefly, DNA was incubated at 55°C overnight in lysis buffer (50 mM NaCl, 10 mM Tris pH 8.0, 1 mM EDTA, pH 8.0) containing 1% SDS, 1% (v/v). b-mercaptoethanol and 2 mg/ml proteinase K. DNA was isolated by phenol/chloroform extraction, were quantify and 10 μg of DNA were loaded in each gel well.

### Histology

Scrotal heat stress, γ-radiated and control mice were killed by cervical dislocation, and the testes were fixed in Davidson Fluid modified [26] for 48 h, then rinsed with PBS, and stored in 70% ethanol until analysis. Entire testes were embedded in paraffin using routine histologic procedures for subsequent light microscopic evaluation. Serial sections measuring 10 μm in thickness were then cut from the paraffin blocks and selected for staining. Ten sections were taken from every mouse testis. Finally, all sections were stained with hematoxylin/eosin. Two investigators blinded to the groups interpreted structural changes. At least 8 animals per treatment were analyzed.

### Statistical analysis

Statistical analyses were performed using SigmaStat version 3.1.1 software (Jandel Scientific, San Rafael, CA). Data are given as the mean ± S.E.M. Comparison of the differences between the means for each treatment were done using ANOVA followed by Student-Newman-Kleus post hoc test.

## Results

### Effect of treatment on morphology of seminiferous tubules and sperm characteristics

DNA fragmentation was induced by scrotal heat stress and γ-radiation. Based on previous results [16] we analyzed the effect of the treatments 21-25 days post heat stress o γ-radiation in order to asses the effect of such insult on the spermatocytes at the time of treatment, one of the more sensitive stages of spermatogenesis, when a higher DNA fragmentation is induced [16,27]. 21-25 days is the estimated time that spermatocytes need to become mature spermatozoa.

Neither heat stress nor γ-radiation treatments had any significant effect on body weight of mice 21 days after treatment. Both treatments reduced testicular weight, number of spermatozoa and motility, but the detrimental effect was significantly higher in the heat stress group compared to the radiated group (Table 1).

**Table 1:** Effects of treatment with heat on body weight, testis weight, epidydimal weight and sperm characteristics - motility and sperm counts (ANOVA analysis)

	Treatment Group		
	Control (n = 9)	Scrotal Heat Stress (n = 8)	γ Radiated (n = 10)
Body weight (g)	37.06 ± 1.69	35.96 ± 0.73	36.72 ± 0.74
Testis weight (mg)	239 ± 14a	138 ± 10b	127 ± 5b
Testis weight per body weight (mg/g)	6.06 ± 0.88a	2.54 ± 0.36b	3.47 ± 0.13b
Epididymal weight (mg)	133.0 ± 10.5	113. 5 ± 10.7	133 ± 27
Vas deferens sperm counts (×106)	11.6 ± 0.8a	4.4 ± 1.7b	7.6 ± 1.3c
Motility (%)	69 ± 3a	21 ± 5b	52 ± 4c

Values with different alphabetical superscripts in each row differ significantly (P < 0.05)

**Figure 2:**
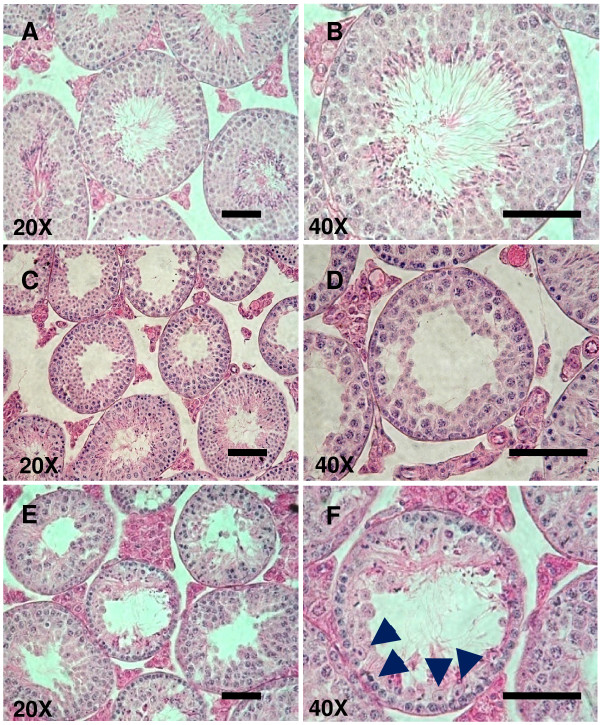
**Histological comparison of seminiferous tubules from testes from control group (A, B); testes 21-25 days after exposition to 42°C, 30-min (scrotal heat stress) (C, D); and testes 21-25 days after γ-radiation treatment (E, F); stained with hematoxylin/eosin**. Bar 50 μm.

Histological examination of testes from control mice showed normal spermatogenesis, with nearly all tubules showing evidence of endogenous spermatogenesis. In testes from both heat stressed or radiated groups, abnormal spermatogenesis and loss of the architecture of seminiferous tubule was observed (Fig. 2). A depletion of cells was noted in both treated groups, with the majority of tubules containing very low number of spermatid and mature spermatozoa. Also, in both treatments, it was observed a disorganization of germinal cells. Pyknotic (chromatin condensation) and necrotic nuclei were retrieved as well. In the radiated group it is observed hyperplasia of Leidig cells between seminiferous tubules (Fig. 2E and 2F).

### Effect of treatment on implantation, resorptions, pregnancies and quality of embryo produced *in vivo*

**Figure 3:**
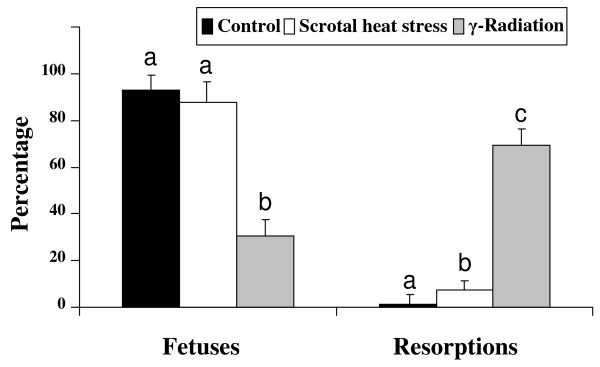
**Mean numbers of implantation sites, day 14 fetuses, and resorption sites per pregnant female after mating with control (n = 13, 13 out 13 pregnant), scrotal heat stressed males (n = 18, 6 out 18 pregnant), and γ-radiated males (n = 12, 12 out 12 pregnant)**. Means ± SEM. Different letters indicate significant differences among treatments (P < 0.05, ANOVA analysis).

A reduction in pregnancy rates was obtained in the heat stressed group; all females of the control group (13 of 13) and of γ-radiated group (12 of 12) became pregnant compared to 33% (6 out 18) of the heat stressed group (Fig 3). We observed that scrotal heat stress but not γ-radiation affected pregnancy rate (Fig 3). Both treatments decreased significantly implantation rates however, number of foetuses conceived after mating with males subjected to γ-radiation (n = 12; 3.83 ± 0.59) was lower than the one observed with the heat stressed (n = 6; 8.67 ± 1.18) or control groups (n = 13; 13.85 ± 0.88). Radiation, but not heat stress, increased significantly resorption rate, indicating that spermatozoa from radiated mice are able to fertilize *in vivo *and to produce blastocysts able to implant, however embryo viability is somehow compromised as it is reflected by the lower proportion of live embryos obtained

### DNA integrity of sperm recovered from uterus, oviduct, and sperm attached to the zona pellucida

Sperm cells were recovered, 21-25 days after treatment, from 3 different regions of the female tract: the isolated oviduct (Ov), the proximal uterine region close to the uterotubal junction (Uo), and the distal uterine region close to the cervix (Uc) (Fig. 1A). Neutral comet assay and alkaline gel electrophoresis (Fig. 1B) were used to evaluate damage in double and single strand of DNA, respectively. Heat stressed mice showed higher levels of DNA damage in spermatozoa recovered from vas deferens (tail length and tail moment) than control or radiated groups (Table 2). In control and treatments groups, sperm DNA fragmentation was higher in the lower and upper uterus than in the oviduct (Table 2). When sperm populations were classified according to their tail length (Additional File 1, Figure S1) or tail moment (Additional File 2, Figure S2), we observed that most of the spermatozoa population collected from the oviduct had low values of tail length and tail moment both in the control and in the treated groups; indicating that spermatozoa with higher motility which are capable to reach the oviduct have lower lever of DNA fragmentation than less motile spermatozoa

**Table 2:** Effect of scrotal heat stress and γ-radiation on Tail length on Comets from spermatozoa recovered from different sections along uterine tract and assessed by neutral Comet assay

Treatment	Tail Length			
	Ep	U_c_†	U_ov_†	Ov†
**Control**	6.71 ± 0.82^aα^	30.94 ± 5.62^b^	25.71 ± 2.42^b^	8.86 ± 0.8^a^
**(n = 9)**				
**Scrotal Heat Stress**	38.96 ± 4.09^aβ^	38.37 ± 4.66^a^	29.39 ± 3.52^a^	11.20 ± 1.50^b^
**(n = 9)**				
**γ-Radiation**	17.20 ± 3.58^aγ^	44.58 ± 12.08^b^	32.38 ± 9.58^ab^	11.18 ± 2.99^b^
**(n = 8)**				

Significant differences in tail length and tail moment from spermatozoa recovered in each section analyzed, within a treatment, were denoted with different Latin alphabetical superscript in each row; and within a section were denoted with different Greek superscripts in each column. Means ± SEM. Values with different superscripts differ significantly (P < 0.05) Ep, epididymal sperm; Uc, sperm population recovered from uterine tract near cervix; Uo, sperm population recovered from uterine tract near oviduct and Ov, sperm recovered from oviduct† Indicates no significant differences among treatments within a section.

For the IVF protocol, sperm cells capacitated for 60 min were collected near the border of the sperm-containing drop of medium. This procedure resulted in the selection of spermatozoa having a higher motility. Two hours after IVF, sperm cells attached to the ZP were analyzed and compared with the non-attached spermatozoa. Spermatozoa attached to the ZP from the heat stress and radiated groups had less Tail Length and Tail Moment than the non attached spermatozoa (Table 3), indicating that those spermatozoa able to attach to the ZP have lower DNA fragmentation than the non attached. This could be due to the higher motility of the sperm with non fragmented DNA and/or to a ZP binding selection of spermatozoa without DNA damage. Comparing spermatozoa attached to the ZP in the control and treated groups, we have observed that Tail Length was higher in the radiated group than in the control, and Tail Moment was higher in both treated groups compared to the control. This indicates that spermatozoa with fragmented DNA fragmented are able to attach to the ZP.

**Table 3:** Effect of scrotal heat stress and γ-radiation on Tail length on Comets from spermatozoa recovered after *in vitro *fertilization from zona pellucida and assessed by neutral Comet assay

Treatment	Tail Length		Tail Moment	
	IVF	ZP†	IVF	ZP†
**Control**	16.20 ± 1.02^aα^	14.76 ± 3.98^a^	4.55 ± 0.56^aα^	3.72 ± 1.30^a^
**(n = 9)**				
**Scrotal Heat Stress**	26.64 ± 3.34^aβ^	15.96 ± 2.92^b^	14.82 ± 2.34^aβ^	8.96 ± 3.31^b^
**(n = 9)**				
**γ-Radiation**	46.95 ± 6.55^aγ^	21.43 ± 5.84^b^	29.90 ± 6.34^aγ^	8.68 ± 3.15^b^
**(n = 8)**				

Significant differences in tail length and tail moment from spermatozoa recovered in each step of IVF, within a treatment, were denoted with different Latin alphabetical superscript in each row; and within an IVF step were denoted with different Greek superscripts in each column. Means ± SEM. Values with different superscripts differ significantly (P < 0.05) Ep, epididymal sperm; IVF, sperm population recovered from *in vitro *fertilization drop; ZP, sperm population attached to Zona Pellucida. † Indicates no significant differences among treatments within ZP.

### Consequences of the use of spermatozoa from treated males for IVF and ICSI in embryo preimplantation development

IVF experiments showed a lower percentage of 2-cell cleavage in the heat stressed group compared to the control group (Table 4). This could be due to the lower motility of the sperm. However, blastocyst development was not different between control and heat stressed groups, indicating that sperm from heat stressed mice that are able to fertilize are no different from control spermatozoa regarding postimplantational development. When γ-radiated mice were analyzed, we found a higher 2-cell cleavage rate than in heat treated group. However, blastocysts development was lower compared to the control and heat stressed groups, indicating that sperm from radiated mice should have some DNA-damage which allows fertilization but compromise embryo development.

**Table 4:** Cleavage rate and development of oocytes fertilized by *In Vitro *fertilization and ICSI with non-treated, scrotal heat stressed and γ-radiated spermatozoa

Technique	Sperm treatment	Total oocytes (experiments)	2-cell embryos (%)		Blastocyst (%)
**IVF**	**Control**	107 (5)	99 (93)^a^		95 (96)^a^
	**Scrotal Heat Stress**	223 (8)	29 (13)^b^		26 (90)^a^
	**γ-Radiation**	85 (5)	56 (66)^c^		36 (63)^b^
	**Sperm treatment**	**Injected Oocytes (experiments)**	**Surviving Oocytes (%)**	**2-cell embryos** **(%)**	**Blastocyst** **(%)**
**ICSI**	**Control**	72 (3)	53 (74)	46 (87)^a^	31 (67)^a^
	**Scrotal Heat Stress**	232 (6)	158 (68)	74 (47)^b^	4 (6)^b^
	**γ-Radiation**	131 (4)	94 (72)	65 (69)^c^	28 (43)^c^

Means ± SEM. Different alphabetical superscript indicates significant differences (P < 0.05, ANOVA analysis) among treatments.

When natural barriers of fertilization were overcome by ICSI, number of surviving oocytes was not affected by the source of spermatozoa (control, heat-stressed or radiated), but both cleavage rate and blastocyst developmental rate of the control group was significantly higher than in the heat stressed and radiated groups, suggesting a lower DNA quality of these spermatozoa.

## Discussion

The female reproductive tract holds the key to mammalian sperm selection. After mating there is a preselection of spermatozoa based on their motility, and those that successfully overcome this selection become sequestered within the oviductal sperm reservoir until ovulation takes place, then spermatozoa are untied and proceed towards the oocytes. When mating takes place after ovulation, spermatozoa only may undergo some selection processes during the transport through the female reproductive tract and/or during the zona pellucida (ZP) binding/penetration, since there is no sperm attachment to the oviduct. The aim of this work was to investigate in a post-ovulatory mating mice model (mating 6-8 hours after ovulation), the selection mechanisms that operate in nature and to find out if these mechanisms can discriminate the quality of spermatozoal DNA regardless the source of damage; a heat stress process, or a gamma radiation process. Our results indicate that in postovulatory mating there is a preliminary general selection mechanism of spermatozoa with undamaged DNA during the transport through the female reproductive tract and in the ZP binding, but the ability of the ZP to recognize fragmented-DNA spermatozoa is achieved during sperm-ZP penetration, and it depends on the source and type of damage, because only DNA-damaged sperm cells by heat stress but not by radiation were selected. The preliminary selection could be a consequence of selection based on motility since DNA fragmentation and sperm motility have been related [7]. Here, we have demonstrated that higher motile sperm cells that reach the oviduct have lower DNA fragmentation that sperm with lower motility present in the uterus. However, sperm with some level of DNA damage is able to reach the oviduct and to attach to the ZP. Based on these findings the sperm-ZP selection during penetration should be a specific mechanism, because it is only active when sperm-DNA has been fragmented by effect of heat stress and it is not when the damage has been produced by radiation. It has been suggested that stable chromatin structure provides sperm with a rigidity that enables them to penetrate the zona pellucida [28].

The damage induced in the testes, and the nature and degree of sperm DNA-damage produced by scrotal heat stress or by γ-radiation seems different and may influence the sperm fertilizing capacity. Heat stress decreased testis weight, sperm counts retrieved from vas deferens and motility as described by other authors [29]. In case of radiation, a decrease of testis weight was also observed, a situation that has been reported by other authors [30]; however, the decrease in number of spermatozoa and sperm motility was less pronounced than with the heat stress. In agreement with previous reports [16,31,32], scrotal heat stress produced changes in testicular architecture and DNA-damage of sperm. Histology of testes indicated similar reduction of spermatogenesis in both treated groups; however, in the radiated group it is observed hyperplasia of Leydig cells that might be a consequence of a supraphysiologic hormonal stimulation of the testes originated by the radiation treatment of the male mouse. At higher doses of radiation, a significant loss of primary spermatocytes has been reported [33]. High-temperature in testis is associated with an increase of oxygen free-radical species, which reacts quickly with substrates as lipids, causing a lipid peroxidation that affects spermatozoa membranes [34]. Next to these effects, mitochondrial membrane can be disrupted in spermatozoa and germ cells [35,36] activating programmed cell death pathways. Gamma radiation tends to cause damage on DNA strands directly (ds or ss breaks) [37], and more specifically, mutations and alterations of DNA backbone (crosslinking among base pairs, presence of apurinic or apyrimidinic sites) [38]. Alterations in DNA backbone could alter level of chromatin condensation and nuclear volume [39], parameters related with susceptibility to damage by radiation (Hawkins, 2005). The assay that we have used for quantifying sperm DNA-fragmentation can not determine this second type of mutations produced by the radiation.

Motility is the main factor that allows the sperm to reach the oviduct through the cervix and the utero tubal junction [40] but it seems that motility is not the unique factor of the sperm selection mechanisms; chemical barriers (e.g., low pH and viscous mucus) and leuckocytic/phagocytotic responses within the female, determine that only a small proportion of the sperm cells deposed in the female tract ever have the opportunity to encounter an oocyte. During some phases of sperm transport through the female reproductive tract, sperms are subjected to physical stresses, are exposed to factors with cell signaling capabilities, and may sustain oxidative damage to their plasma membrane lipids. The initial stages of sperm transport are also mediated by the female tract, because sperm transport is regulated by a combination of intrinsic sperm motility and peristaltic movements of the female reproductive tract. We have found that sperm DNA fragmentation level in spermatozoa recovered from uterus increased compared to sperm DNA fragmentation observed in vas deferens spermatozoa, both in control and in gamma radiated groups. In heat-stressed group, sperm DNA fragmentation in the uterus was also high, but not higher than the value detected for vas deferens spermatozoa. These observations indicate that some damage in sperm DNA is taking place in the uterus. Two mechanisms could be responsible of this effect. On one hand, immune cells present in the uterine mucosa [41-43] such as neutrophils could extrude their nuclear DNA next to associated proteins to form neutrophil extracellular traps (NETs) [44] that retain spermatozoa with disturbed motility or plasma membrane. During NETs disaggregation, DNAses present in the seminal plasma [45,46] can act removing connections between neutrophils, attached sperm and proteins, allowing an efficient cleaning of the reproductive tract. The second mechanism could be the presence of nucleases from seminal fluid affecting spermatozoa located in the uterus [46,47].

Our results agree with previous reports indicating that the passage thought the utero-tubal junction *in vivo*, is one of the most important selective points that are involved in a plausible selection mechanism in which motility plays a main role [10]. In addition, we have found that the transit form the uterus to the oviduct is critical to reduce sperm with fragmented-DNA. Spermatozoa from the three groups analyzed arriving at the oviduct showed drastic reduction in DNA-damage. This would indicate that there is a selection mechanism in the female reproductive tract based on sperm motility but that indirectly, ensures that spermatozoa with the most intact DNA arrive to the oviduct probably a consequence of a relationship between motility and DNA-fragmentation. We have analyzed the DNA-fragmentation by COMET of high motile sperm selected *in vitro*, taking the faster population of spermatozoa that are able to cross thought drops of media and we have found a correlation between high motility and low level of DNA-fragmentation (data non published). Other authors have also suggested this relation between motility and DNA-fragmentation by *ex vivo *or *in vitro *approaches [7,48]. However, we have found that *in vivo*, fertilization with radiated sperm produced a higher number of resorptions, suggesting that in radiated sperm, several DNA-damages that are carried by motile sperm are selected neither in the female tract nor in the ZP penetration. Collectively, these results suggest that there is a relationship between motility and the sperm with damage on DNA strands (ds or ss breaks), but not with mutations and/or alteration of DNA backbone (crosslinking among base pairs, presence of apurinic or apyrimidinic sites).

To analyze if the ZP binding and/or penetration plays a role in the selection of sperm with fragmented-DNA, we have examined the DNA quality of sperm cells attached to the ZP and the preimplantation development of oocytes fertilized *in vitro *or by ICSI. Sperm cells from treated mice that are attached to the ZP exhibits lower levels of DNA fragmentation that the non-attached sperm, indicating that either ZP performs a positive selection towards unfragmented DNA sperm or that motile sperm (with lower level of fragmented-DNA) are more capable to attach to the ZP. This agrees with previous findings demonstrating that a high number of spermatozoa with normal chromatin were attached to ZP [13]. Samples taken from the fertilization drop showed that there is an increase in sperm DNA fragmentation levels compared to the levels of fragmentation observed in the vas deferens of the control and radiated groups (data not shown). This fact could be explained by the presence of endonucleases delivered from dead sperm cells during IVF [49-51]. Some reports have suggested that membrane-altered spermatozoa liberate some factors to the media, including endonucleases, that could fragment DNA of spermatozoa with unaltered membranes [52]. Also, an increase in ROS production during IVF could take place, escalating damage on alive spermatozoa [53]. In addition, IVF experiments showed that sperm form heat stressed mice that are able to fertilize are also able to produce blastocysts, but when the spermatozoa attached to the ZP are delivered by ICSI, blastocysts development is reduced, indicating that the ability of the ZP to select fragmented-DNA spermatozoa is achieved during sperm-ZP penetration. In the case of sperm from radiated mice, blastocyst development is reduced in comparison to control both after IVF or ICSI, indicating that the ZP is unable to select sperm with DNA damage induced by radiation. These findings agree with our *in vivo *results since many of the blastocysts produced, apparently normal, could implant, but failed to carry on a normal foetal development and were reabsorbed. On the other hand, *in vivo *results with heat stressed mice indicate that resulting blastocysts after mating are as competent as control blastocysts to develop into live foetuses. Our results of IVF from heat stress males differ from other study where a block of embryonic development was detected using C57BL/6 inbred mice [32]. The differences may be due to the strain of mice, because we have used CD1 outbreed mice, and it is well established that the genetic background of outbreed and inbred strains of mice influence sperm-assessment parameters, resistance and susceptibility to DNA fragmentation, *in vitro *fertilization rate, and *in vitro *embryo development rate; moreover, sperm from the C57BL/6 backgrounds is particularly sensitive [54]. For this reason, results from some specific inbred strains should be considered with precaution.

## Conclusion

Our results indicated that when mating takes place after ovulation, female reproductive tract and ZP binding/penetration process play an important role not only in selection of sperm with normal motility and morphology, but also with normal chromatin DNA and/or normal DNA structure/function. The selections in the female tract and during ZP-binding are related to the high correlation between normal motility and normal chromatin DNA. Moreover, there is an active mechanism of selection for sperm with unfragmented DNA that is taking place during the ZP-penetration; however, this mechanism can not recognize sperm carrying some DNA abnormalities, as the DNA mutations originated by radiation, suggesting that other damages concomitantly to DNA fragmentation might be triggered by heat stress, but not by radiation, and thus selection should be based on sperm phenotype and/or function associated with these damages. If the selection mechanisms that operate in nature are able to discriminate the quality of spermatozoa, understanding the basis of the naturally imposed selection mechanisms may help to clarify, which of the many laboratory test are likely to be most informative about fertility. This is of crucial importance in certain situations for example when ICSI treatments are performed as assisted reproductive procedure, because ICSI bypasses multiple natural mechanisms, apparently redundant, which have evolved to ensure selection of high quality sperm cells for fertilization [24]. Sperm DNA fragmentation seems to affect embryo post-implantation development in ICSI procedures in human, resulting in pregnancy loss [55]. Our results strongly suggest that for some types of DNA damage, IVF is a preferential system than ICSI, and that ICSI should be performed with high motile sperm to ensure fertilization with less fragmented-DNA spermatozoa.

## Abbreviations

Ov: oviduct; Uo: uterus near oviduct; Uc: uterus near cervix; ZP: zona pellucida; IVF: *In Vitro *fertilization; ICSI: Intracytoplasmic Sperm injection.

## Competing interests

The authors declare that they have no competing interests.

## Authors' contributions

JDH, MPC, and RFG performed the research. JDH and AGA designed the study. JDH, BP, and AGA interpreted the results and drafted the manuscript. All authors read and approved the final manuscript.

## Supplementary Material

Additional file 1 Supplemental Figure 1. Percentage of sperm with DNA-damaged expressed as the Comet assay Tail length in spermatozoa recovered from female reproductive tract. Ep, epididymal sperm; Uc, sperm population recovered from uterine tract near cervix; Uo, sperm population recovered from uterine tract near oviduct and Ov, sperm recovered from oviduct. Click here for file

Additional file 2 Supplemental Figure 2. Percentage of sperm with DNA-damaged expressed as the Comet assay Tail moment in spermatozoa recovered from female reproductive tract. Ep, epididymal sperm; Uc, sperm population recovered from uterine tract near cervix; Uo, sperm population recovered from uterine tract near oviduct and Ov, sperm recovered from oviduct. Click here for file
