# Supplementary figures and images for: Selection against spermatozoa with fragmented DNA after postovulatory mating depends on the type of damage
Source: Reprod Biol Endocrinol. 2010 Jan 31;8:9. doi: 10.1186/1477-7827-8-9 (PMC2825232; doi:10.1186/1477-7827-8-9)

## Slide 1
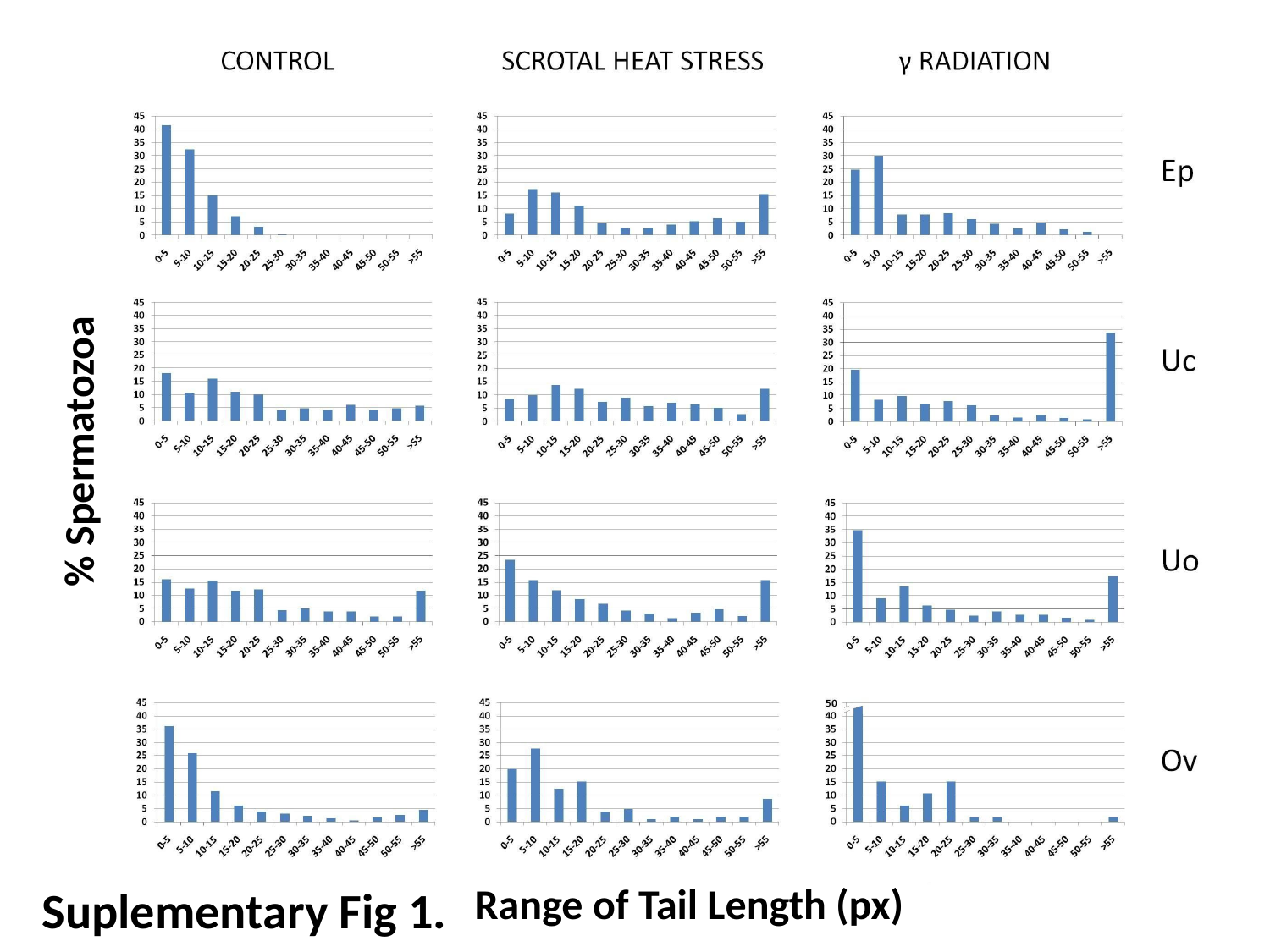

% Spermatozoa
Range of Tail Length (px)
Suplementary Fig 1.

Supplement: Additional file 1 — Supplemental Figure 1. Percentage of sperm with DNA-damaged expressed as the Comet assay Tail length in spermatozoa recovered from female reproductive tract. Ep, epididymal sperm; Uc, sperm population recovered from uterine tract near cervix; Uo, sperm population recovered from uterine tract near oviduct and Ov, sperm recovered from oviduct. [file 1477-7827-8-9-S1.PPT]

## Slide 1
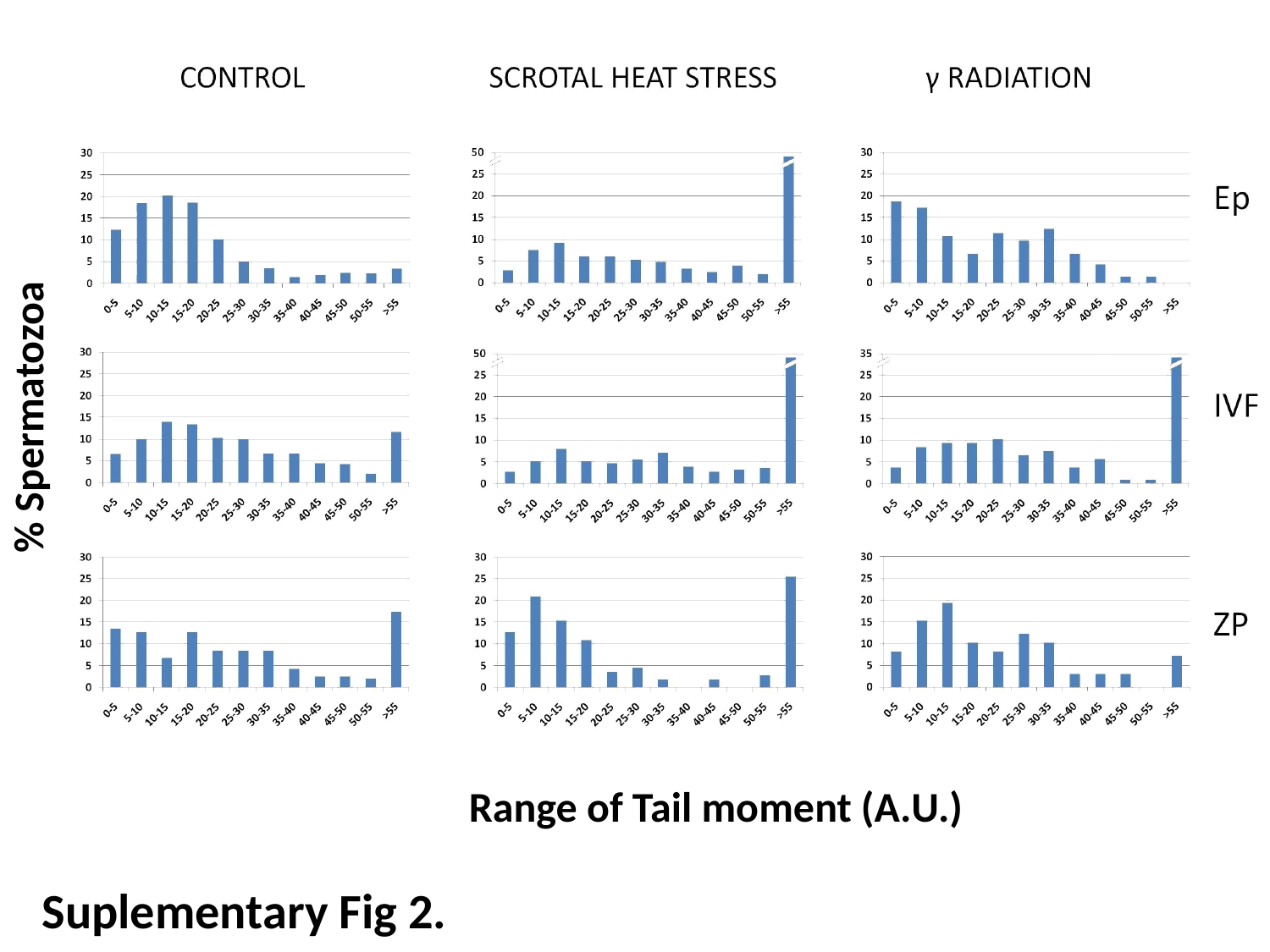

% Spermatozoa
Range of Tail moment (A.U.)
Suplementary Fig 2.

Supplement: Additional file 2 — Supplemental Figure 2. Percentage of sperm with DNA-damaged expressed as the Comet assay Tail moment in spermatozoa recovered from female reproductive tract. Ep, epididymal sperm; Uc, sperm population recovered from uterine tract near cervix; Uo, sperm population recovered from uterine tract near oviduct and Ov, sperm recovered from oviduct. [file 1477-7827-8-9-S2.PPT]
